# Potential Contribution of Short Chain Fatty Acids to Hepatic Apolipoprotein A-I Production

**DOI:** 10.3390/ijms22115986

**Published:** 2021-06-01

**Authors:** Herman E. Popeijus, Willem Zwaan, Jehad Z. Tayyeb, Jogchum Plat

**Affiliations:** 1 Department of Nutrition and Movement Sciences, NUTRIM School for Nutrition and Translational Research in Metabolism, Maastricht University, 6229 ET Maastricht, The Netherlands; willemzwaan@hotmail.com (W.Z.); j.tayyeb@maastrichtuniversity.nl (J.Z.T.); j.plat@maastrichtuniversity.nl (J.P.); 2 Department of Clinical Biochemistry, Faculty of Medicine, University of Jeddah, Jeddah 23218, Saudi Arabia

**Keywords:** cholesterol metabolism, reverse cholesterol transport, short chain fatty acids, high-density lipoprotein, Apolipoprotein-A-I

## Abstract

Apolipoprotein A-I (ApoA-I) is the major protein of high density lipoprotein (HDL) particles and has a crucial role in reverse cholesterol transport (RCT). It has been postulated that elevating production of de novo ApoA-I might translate into the formation of new functional HDL particles that could lower cardiovascular disease (CVD) risk via RCT. During inflammation, serum ApoA-I concentrations are reduced, which contributes to the development of dysfunctional HDL particles as Serum Amyloid A (SAA) overtakes the position of ApoA-I within the HDL particles. Therefore, instead of elevating serum HDL cholesterol concentrations, rescuing lower serum ApoA-I concentrations could be beneficial in both normal and inflamed conditions. Several nutritional compounds, amongst others short chain fatty acids (SCFAs), have shown their capacity to modulate hepatic lipoprotein metabolism. In this review we provide an overview of HDL and more specific ApoA-I metabolism, SCFAs physiology and the current knowledge regarding the influence of SCFAs on ApoA-I expression and synthesis in human liver cells. We conclude that the current evidence regarding the effect of SCFAs on ApoA-I transcription and secretion is promising, however there is a need to investigate which dietary fibres could lead to increased SCFAs formation and consequent elevated ApoA-I concentrations.

## 1. Introduction

Cardiovascular disease (CVD) is currently the leading cause of deaths worldwide [1,2]. As of 2017, CVD results in approximately 17.9 million deaths each year, representing 31% of all worldwide deaths [3]. Amongst other risk factors, CVD risk profiles are characterized by prolonged dyslipidemia, which leads to progressive atherosclerosis [4]. Although lowering serum low-density lipoprotein cholesterol (LDL-C) concentrations successfully lowers CVD development, there remains a notifiable residual risk [5]. To further reduce this remaining risk, increasing serum high-density lipoprotein cholesterol (HDL-C) concentrations was thought being a promising strategy to prevent CVD for a long time. However, this paradigm no longer holds. Large scale intervention studies that elevated serum HDL-C concentrations failed to lower CVD development [6,7]. This resulted in the development of alternative explanations how to position the link between the HDL-C fraction and CVD risk, what we nowadays call “functional HDL-C”. This implies that it is not simply elevating HDL-C concentrations that is protective but increasing the functionality of HDL particles that makes them able to fulfil a protective role. In this context, Apolipoprotein A-I (ApoA-I) is an important player as it is the major protein of the HDL-C fraction. There is ample evidence that administrating ApoA-I protein into the bloodstream results in the formation of new small HDL particles, improves the lipid profile [8], and lowers the CVD risk [7]. Unfortunately, ApoA-I concentrations are reduced under inflammatory conditions. These inflammatory conditions coexist under CVD and as consequence further potentiate dyslipidemia contributing to an increased risk of CVD development [9]. Therefore, increasing the endogenous production of ApoA-I protein and consequently supplying the serum with fresh empty HDL particles, especially under inflammatory conditions, seems warranted to lower CVD risk and counter dyslipidemia. 

Several diet-derived components have been shown to modulate lipid and lipoprotein metabolism [10]. One group of components that has been described to show these effects are the short chain fatty acids (SCFAs). SCFAs are not only a substrate in lipid metabolism but are also involved in its regulation [11]. It has been well characterized that dietary fibre is the substrate for intestinal microbiota mediated production of SCFAs [12,13]. Thus, consumption of products high in specific dietary fibres leads to increased intestinal SCFAs production, which results in multiple health benefits such as an improved lipid profile and counteracting diseases like type 2 diabetes, cancer, and CVD [13,14]. In addition, SCFAs have shown to increase both HDL and ApoA-I concentrations in cell as well as in animal studies [15,16]. Mechanistically, SCFAs seem to play a role in the production of ApoA-I which could consequently improve the functionality of the serum HDL fraction [16]. In this review, we present the current evidence regarding the influence of SCFAs on hepatic ApoA-I transcription and production under normal and metabolically disturbed conditions such as inflammation.

## 2. ApoA-I as Interesting Target to Modulate HDL Metabolism

Lipoprotein metabolism and cholesterol homeostasis are complex and tightly regulated processes in which HDL particles play an important role. HDL-C is considered as the healthy type of cholesterol, as studies have established an association between low plasma HDL-C concentration and an elevated risk of cardiovascular diseases [17]. ApoA-I is the main structural protein of HDL, accounting for 70% of the HDL particles [18]. ApoA-I has a critical role in HDL-C assembly and acts as the acceptor of cholesterol from peripheral cells since it is the ligand for the ATP binding cassette transporters ABCA1 and ABCG1. The liver is responsible for most of the production and secretion of ApoA-I (70%), whereas the remaining (30%) is secreted by the small intestine [19]. Despite the cross-sectional inverse relation between HDL-C and CVD risk, increasing serum HDL-C concentrations was unexpectedly not associated with a reduced CVD risk [20]. More recently, HDL particle size and its functionality were postulated as the most important factors instead of solely the plasma HDL-C concentration in preventing the development of atherosclerosis and CVD [21,22,23]. 

**Figure 1:**
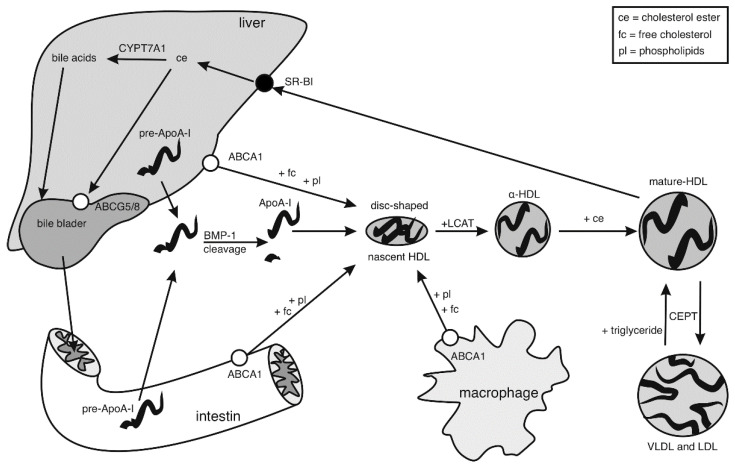
Overview of the high density lipoprotein (HDL) and Apolipoprotein A-I (ApoA-I) metabolism in both liver and intestine. In the plasma, lipid-free pro-ApoA-I is cleaved by bone morphogenetic protein-1 (BMP-1) to form ApoA-I and then interacts with ATP-binding cassette subfamily A member 1 (ABCA1) which mediates cholesterol and phospholipid efflux to lipid-poor ApoA-I, forming a disc-shaped nascent HDL particle. Additionally, nascent HDL can take up phospholipids and free cholesterol from peripheral locations, mainly macrophages. Nascent HDL readily transforms into α-HDL as it interacts with lecithin cholesterol acyltransferase (LCAT) followed by cholesterol esterification and HDL maturation. Mature HDL exchanges nuclear cholesterol esters with triglyceride from low-density lipoprotein (LDL) and very-low-density lipoprotein (VLDL) particles via ester transfer protein (CETP), resulting in triglyceride-rich HDL particles. HDL particles dock to scavenger receptor class B member 1 (SR-BI), which regulates the transport of cholesterol esters out of the HDL particles into the cells. Then, hepatic cholesterol can be metabolized into bile acids by cholesterol 7 alpha-hydroxylase (CYP7A1) or excreted directly into bile through ATP-binding cassette sub family G members 5 and 8 (ABCG5 and ABCG8).

HDL particles not only carry cholesterol but they are involved in multiple biological processes, such as the transportation of micro RNAs and other cargo such as specific proteins, carotenoids, and hormones [24]. Additionally, they have been shown to possess anti-inflammatory and anti-thrombotic effects [18,24]. Nevertheless, the most important function, which mainly explains their athero-protective properties, is the role of HDL particles in the reverse cholesterol transport (RCT) process (Figure 1). RCT starts when pro-ApoA-I is excreted to the plasma where bone morphogenetic protein-1 (BMP-1) converts it into mature ApoA-I [25], then the lipid-free mature ApoA-I interacts with the ATP-binding cassette subfamily A member 1 (ABCA1) [18]. This mediates cholesterol and phospholipid efflux to lipid-poor ApoA-I, forming a disc-shaped nascent HDL particle stabilized by two ApoA-I molecules [17]. After that, nascent HDL readily transforms into spherical HDL (α-HDL) as it interacts with liver synthesized lecithin cholesterol acyltransferase (LCAT) [26]. Next, LCAT converts cholesterol into cholesteryl ester, causing cholesterol esterification and HDL maturation [18]. The mature HDL exchanges nuclear cholesterol esters with triglyceride from LDL and VLDL cholesterol via the ester transfer protein (CETP), resulting in triglyceride-rich HDL particles. Then, in the direct RCT, HDL particle dock to the scavenger receptor class B member 1 (SR-BI), which regulates the transport of cholesterol esters out of the HDL particles into the cells [18,27]. In the indirect pathway, the HDL particle transfers its cholesteryl esters to potentially atherogenic LDL and IDL particles via CETP. Both cholesteryl ester and free cholesterol content within these particles are taken up by the liver via the low-density lipoprotein receptor (LDL-R), which binds the Apolipoprotein B-100 (ApoB-100) component of these lipoproteins [9,28,29]. Finally, both direct and indirect RCT pathways result in the transfer of cholesterol from peripheral locations, mainly macrophages, to the liver. At this point, the hepatic cholesterol can either be redistributed to peripheral tissues when it is secreted in VLDL particles, metabolized into bile acids by cholesterol 7 alpha-hydroxylase (CYP7A1), or excreted directly into bile through ATP-binding cassette sub family G members 5 and 8 (ABCG5 and ABCG8) [29]. To summarize, during the RCT, the HDL particles transfer cholesterol from peripheral tissues back to the liver. Consequently, cholesterol will either be redistributed within the human body or excreted via the bile [9,18]. As a result of RCT, less cholesterol will remain in the bloodstream, which ultimately reduces atherosclerotic plaque formation and positively affects the cardiovascular health. In this process, newly synthesized ApoA-I is crucial since it is the start for the synthesis of fresh HDL particles ready to take-up excess of cholesterol through ABCA-I mediated cholesterol efflux from macrophages to the HDL fraction, and therefore essential for a functional HDL pool [30].

## 3. Production, Absorption, and Functions of SCFAs

Dietary fibres and resistant starch are known to beneficially influence a range of metabolic parameters, including controlling blood glucose profiles and lowering serum cholesterol concentrations [13,31]. Recent studies revealed that these beneficial health effects associated with high-fibre diets are (at least partly) attributed to the formation of SCFAs [32,33,34]. SCFAs, the end products of gut microbial fermentation of indigestible dietary components, are taken up preferentially via the portal vein and transported to the liver and consequently also increase circulating peripheral SCFAs concentrations, although to a limited extent [32,35]. 

SCFAs are a group of fatty acids with a maximum chain length of six carbon atoms, including formic acid (C1), acetic acid (C2), propionic acid (C3), butyric acid (C4), valeric acid (C5), and hexanoic acid (C6) [36]. The most predominantly produced SCFAs in the human intestinal tract are acetate (acetic acid), followed by propionate (propionic acid) and butyrate (butyric acid), accounting for >95% of the total SCFAs production [32,37]. The remaining 5% consists of formate, valerate, and hexanoate. The majority of SCFAs are produced in the caecum and proximal colon [38]. However, in the small intestines SCFAs are also produced though in much lower quantities [38]. In the intestinal lumen, values of SCFAs are found between 2–30 mM, more into detail, acetate (10–20 mM) in the higher range and propionate and butyrate in the lower range (1–5 mM) [39]. Absorption of SCFAs is quick and efficient, resulting in an excretion of only 5–10% of the SCFAs in the faeces [38,40]. SCFAs absorption is processed at the apical membrane of mainly colonic epithelial cells via multiple mechanisms. First, the non-ionic diffusion of protonated SCFAs. Second, the exchange of SCFAs with bicarbonate in a 1 to 1 ratio. Third, the co-transportation with cations via the hydrogen-coupled monocarboxylate transporters 1 (MCT1), MCT2, and MCT4 [13,41,42,43]. Finally, SCFAs can be absorbed via sodium-coupled monocarboxylate transporter 1 (SMCT1) [32,44,45]. Moreover, following absorption of SCFAs into the enterocytes of the caecum, ascending colon and transverse colon, they are drained into the superior mesenteric vein and via the portal vein transported to the liver [32]. SCFAs absorbed in the descending and sigmoid colon are drained into the inferior mesenteric vein and also reach the liver via the portal vein [46]. Bloemen et al. did investigate the concentrations of SCFAs in the portal vein in patients that needed to undergo surgery. They detected concentrations of approximately 22, 6.5, and 5 mM for acetate, propionate, and butyrate, respectively [46]. However, these patients were fasted for ±12 h and received both antibiotics and sedatives prior to surgery. Therefore, it remains unclear what concentrations represent the normal physiological situation, though it is likely higher than the values currently reported.

Once SCFAs are absorbed, there are three major sites where they are metabolized. Firstly, colonocytes use SCFAs (mainly butyrate) as a major energy source, providing approximately 60–70% of the total energy needs [47]. Secondly, butyrate and propionate are metabolized by hepatocytes for gluconeogenesis. Finally, while the majority of acetate (50–70%) is also taken up by the liver, the residual acetate is oxidized by the muscle cells for energy generation [38,48]. Besides being utilized for energy, SCFAs play a role in multiple physiological processes such as regulation of energy production and it positively regulates lipid profiles [49]. Additionally, they affect various aspects of glucose metabolism and appetite and as such positively affect cardio-metabolic profiles [50]. Overall, SCFAs seem to play a key role in the regulation of metabolic health and can be used as potential natural (dietary) compounds in the prevention and management of diseases including CVD [38]. Moreover, SCFAs are also reported to play a positive role in countering obesity-induced chronic low-grade inflammation by activating the anti-inflammatory regulatory T cells and supress pro-inflammatory cytokines [51]. In addition, acetate, propionate and butyrate might directly decrease the release of pro-inflammatory cytokines from the adipose tissue, and as consequence the improved adipose tissue function might reduce ectopic fat storage in important metabolic tissues such as the liver and skeletal muscle [32,51].

## 4. Potential Effects of SCFAs on ApoA-I Metabolism

Besides the variety of well-known beneficial effects of SCFAs as described above, there are also indications that SCFAs affect HDL metabolism. It has for example been shown that SCFAs increase serum HDL-C concentrations in rodents which could be interpreted as a beneficial effects in the context of RCT and CVD risk reduction [15]. However, as explained, elevating HDL-C concentrations only does not, by definition, lower CVD risk. An important question is how this elevation in serum HDL cholesterol concentrations can be explained, so what are the responsible underlying mechanistic pathways? In 1991, Kaptein et al. stated that SCFAs can regulate the ApoA-I synthesis and secretion in the hepatocytes [52]. They showed that butyrate (2 mM) increased ApoA-I secretion in human hepatoma cell line (HepG2) by 2.4-fold after 48 h. Additionally, they postulated that this effect was accomplished via (post) transcriptional regulation since *ApoA-I* mRNA expression also increased 2.3-fold. Most important, Apolipoprotein B100 mRNA expression and protein secretion, another hepatic Apolipoprotein, but part of VLDL and LDL particles, was not changed. This indicates that butyrate specifically enhanced ApoA-I secretion. Interestingly, effects of butyrate exposure on hepatic ApoA-I secretion were time and dose-dependent [53]. Although butyrate was the most potent inducer, also propionate (28%) and valerate (73%) significantly increased ApoA-I secretion while acetate did not. In line with these studies by Kaptein et al., Malle et al. confirmed in HUH-7 hepatoma cells that 2 mM butyrate increased the intracellular ApoA-I protein synthesis by three-fold after 48 h, whereas ApoA-I secretion was 2.5 times higher compared to the control [52,53,54]. More recently, Tayyeb et al. showed that not only butyrate but also other SCFAs resulted in a dose-dependent stimulation of *ApoA-I* transcription after 48 h in HepG2 cells [16]. However, despite the increased *ApoA-I* mRNA expression, ApoA-I protein secretion by these HepG2 cells was not changed. Regarding this unexpected observation, it must be mentioned that Tayyeb et al. cultured the HepG2 without FBS serum, which contains the BMP-1 enzyme that converts pro-ApoA-I to mature ApoA-I, so theoretically it is possible that the protein was secreted but not recognized in the assay [25]. To test the mechanisms underlying these SCFAs effects, changes in the mRNA expression of *kelch-like ECH-associated protein 1 (KEAP1)*, *carnitine palmitoyltransferase 1 (CPT1)*, and *peroxisome proliferator-activated receptor alpha (PPARα)*, as well as the PPARα transactivation, were tested. KEAP1 is a target gene of bromodomain and extra-terminal (BET) and therefore an indication for BET inhibition which is known to increase ApoA-I expression [16]. For the PPARα transactivation, the expression of the target genes CPT1 and PPARα were used. SCFAs treatment showed a decrease in *KEAP1* mRNA transcription while PPARα transactivation, using a PPRE luciferase construct, *PPARα,* and *CPT1* mRNA transcriptions all showed an increase. This indicated that underlying pathways that could be involved in the increase in *ApoA-I* transcription are both BET inhibition and PPARα mediated gene expression. In contrast to these effects in human liver cell lines, Fungwe et al. used primary rat hepatocytes and showed that exposure to 1 mM butyrate for 22–24 h did not have an effect on both ApoA-I synthesis and secretion [55]. The question is how these discrepancies can be explained. Fungwe et al. incorporated 35S labeled methionine into immune-perceptible ApoA-I in rat hepatocytes for the protein determination. This indicates that both the differences in analytical techniques and cell-type might explain these differences in outcomes. Another ApoA-I regulatory route that has been suggested more recently in the context of ApoA-I synthesis is the mechanistic target of rapamycin complex 1 and 2 (mTORC1/2), another key regulator involved in lipid metabolism [56]. However, to the best of our knowledge, MTORC has not been studied in the context of SCFA mediated effects on ApoA-I.

Besides in hepatocytes, ApoA-I is also produced in enterocytes of the small intestine [38]. Marcil et al. showed in differentiated human colon adenocarcinoma (Caco-2) cells, which is a model for small intestinal enterocytes, that butyrate significantly lowered ApoA-I synthesis by approximately 32% [57]. In more detail, the Caco-2 cells were treated with 20 mM butyrate for 20 h. This finding was later more or less confirmed by Tayyeb et al. who showed that in contrast to their effects in HepG2 cells, butyrate treatment (1–6 mM) did not change the *ApoA-I* gene expression in Caco-2 cells [58]. Even in a more sophisticated experimental model using transwells, when Caco-2 cells were co-cultured with HepG2 cells, adding butyrate to the apical side of Caco-2 cells did increase *ApoA-I* transcription in the HepG2 cells that were cultured in the basolateral compartment, but not in the Caco-2 cells. This apparent increase in *ApoA-I* mRNA expression in the HepG2 cells in the lower compartment was less pronounced compared to butyrate treatment directly to the HepG2 cells, and for that a lower bioavailability of the SCFAs might be a logical explanation as the SCFA are likely to be utilized by the Caco-2 cells.

### 4.1. Inflammation and ApoA-I

Since ApoA-I is a negative acute phase protein, which means that concentrations are highly sensitive to inflammation and will severely decrease, it is interesting to examine which are the effects of SCFAs on *ApoA-I* transcription under inflammatory conditions. Tayyeb et al. indeed evaluated the effects of SCFAs on inflammatory pathways in relation to *ApoA-I* transcription in HepG2 cells. Again, dose-response studies with SCFAs were performed but now under both normal and inflamed conditions, mimicked by the absence or addition of inflammatory cytokines [59]. As expected, inflammation significantly lowered *ApoA-I* transcription and all SCFAs, except hexanoic acid, increased *ApoA-I* mRNA transcription even under inflamed conditions. In other words, SCFAs might have the capacity to rescue the inflammation induced *ApoA-I* reduction. To understand the underlying mechanisms as to how SCFAs rescued the reduction in *ApoA-I* mRNA production during inflammation, Tayyeb et al. again evaluated the effects of butyrate on the BET and PPARα pathways. Butyrate dose-dependently increased the *KEAP1* mRNA expression in the inflamed condition while it reduced *KEAP1* mRNA expression in the normal condition. This implies that KEAP is not likely responsible for the increase in ApoA-I during inflammation. Next, *CPT1* mRNA expression dose-dependently increased in both normal and inflamed condition, suggesting that PPARα activation could be linked. Additionally, nuclear factor kappa B (NF-κB) transactivation, Interleukin-8 (IL-8) concentrations, and activator protein 1 (AP-1) expression were analyzed to evaluate changes in the inflammatory pathway. Butyrate significantly lowered NF-κB transactivation in cells transfected with NF-κB in the inflamed conditions. Moreover, in the inflamed condition, propionate, butyrate, and valerate (but not hexanoic acid) also significantly decreased IL-8 secretion. Finally, the AP-1 pathway was evaluated by analyzing potential changes in *c-Fos* and *c-Jun* mRNA expression. When AP-1 is involved, these expressions should be lower. However, a significant reduction was not seen, and therefore Tayebb et al. stated that the AP-1 pathway was probably not involved in the anti-inflammatory effects of SCFAs that elevated *ApoA-I* mRNA expression [59]. Therefore, it can be concluded that propionate, butyrate, and valerate have the capacity to rescue *ApoA-I* transcription under NF-kB mediated inflammatory conditions and PPARα activation (but not KEAP and AP-1) is likely to be involved. The link between SCFAs, inflammation, ApoA-I and atherosclerosis has been evaluated by others as well. For example, Bartolomaeus et al. have shown that SCFAs can decrease atherosclerosis in experimental animal models after propionate treatment [60]. Furthermore, Malle et al. tested the effect of inflammation on ApoA-I parameters by inducing inflammation via adding interleukin 1α (IL-1α), IL-6, or a combination of both to the human HUH-7 hepatoma cells. Next, the influences of IL-1α combined with IL-6 on the ApoA-I synthesis and secretion were measured with and without the addition of 2 mM butyrate. There were no significant differences in both ApoA-I measures between inflamed and normal conditions. These effects are seemingly in contrast to the observations by Tayyeb and co-workers, however the dose of 2 mM was relatively low while Tayyeb et al. used a wider dose dependence range from 0.5–7.0 mM, as the SCFAs showed slight variations in their effectiveness based on their doses. Additionally, different hepatocyte cell lines and other cytokines were used to mimic the inflammatory condition, and together these differences might explain the discrepancies.

These previous studies treated the liver cells directly with the SCFAs, while in normal physiology SCFAs are the end products of indigestible dietary components, fermented by the gut microbiome. So in vivo, SCFAs are produced by in the intestinal lumen, and largely metabolized by the intestinal cells before they are transported to the liver cells. Therefore, Tayyeb and co-workers decided to use a more complex model that mimicked the physiological situation [58]. They investigated the influence of SCFAs on ApoA-I in a trans-well model in which a co-culture of both Caco-2 and HepG2 cells were used to mimic the intestine–liver interaction with and without adding cytokines again to mimic the inflammatory condition. Adding the cytokines to either the apical, basolateral, or to both cell compartments significantly lowered *ApoA-I* mRNA levels in both HepG2 and Caco-2cells. Interestingly, this reduced *ApoA-I* mRNA level in the inflamed HepG2 cells was rescued by providing butyrate to the apical surface of the Caco-2 cells indicating that luminal butyrate is able to affect *ApoA-I* transcription in inflamed HepG2 cells. Increasing butyrate concentrations did not show significant effects on the lowered *ApoA-I* mRNA expression in the presence of cytokines in Caco-2 cells in either apical, basolateral, or both compartments. The remaining question was whether this effect could be attributed to a lower bioavailability or a cross-talk between both intestine and liver cells. Predominantly the effects of butyrate are investigated, especially in combination with inflamed conditions. Therefore, research should also focus on other SCFAs as propionate, valerate, and hexanoic acid are also able to increase *ApoA-I* mRNA expression in both normal and inflamed conditions. 

### 4.2. Antibiotics and ApoA-I

**Figure 2:**
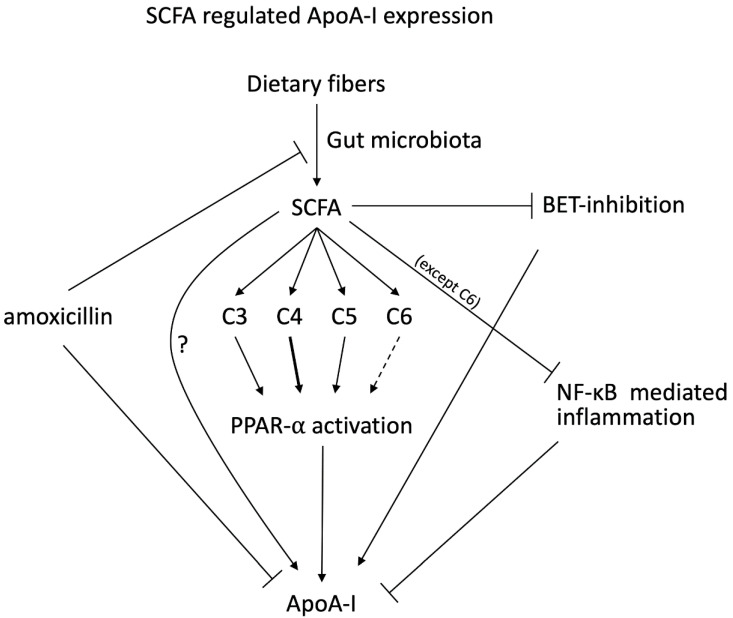
Schematic summary of the review. Short chain fatty acids (SCFA), especially butyrate (C4), is able to increase *Apolipoprotein A-I (ApoA-I)* mRNA concentrations in human hepatoma cell line (HepG2) cells mediated via peroxisome proliferator-activated receptor alpha (PPARα) activation. In addition, SCFAs are able to reduce the effects of inflammation including nuclear factor kappa B (NF-κB) on *ApoA-I* mRNA expression and inhibit the bromodomain and extra-terminal (BET) inhibition. Amoxicillin inhibits both *ApoA-I* mRNA and SCFAs production while the direct effects of SCFAs on ApoA-I still needs to be explored further.

Antibiotics are commonly used drugs for bacterial infections and are particularly effective against gram-negative bacteria, which are the major group with fermentative capacities within the human microbiota [47]. Therefore, antibiotics, as a side effect, also modulate the gut microbiota and alter the presence and expression of several genes as well as derived metabolites such as SCFAs [61]. Multiple studies have investigated a possible effect of antibiotics on lipid metabolism [61,62]. Desmet et al. observed in a placebo-controlled trial that amoxicillin treatment for a duration of 7 days is able to lower serum HDL-C concentrations of healthy participants [61]. Unfortunately, ApoA-I concentrations have not been reported but it is likely that the HDL-C reduction could be attributed to a lower ApoA-I production. This could either be a direct effect of antibiotics on de novo ApoA-I production or indirect via changes in the microbiota composition, which influences the SCFAs production. Previously, Reijnders et al. have shown that effects of vancomycin intake can result in a decreased bacterial diversity in the microbiota, which resulted in a decrease in SCFAs concentrations in the circulation [61]. As Tayyeb and others earlier showed the positive effects of SCFAs on *ApoA-I* transcription together with the observation of reduced SCFAs after antibiotic treatment makes that an indirect effect on HDL-C concentrations via reduced *ApoA-I* transcription is most likely involved. However, ApoA-I expression could also be altered as a direct result of antibiotic treatments, so without the interference of changed SCFAs concentrations. Therefore, Tayyeb et al. explored the possible effects of antibiotics on the ApoA-I transcription and expression [63]. For these experiments, HepG2 cells were cultured without the standard penicillin-streptomycin mixture in the medium to prevent potential bacterial growth. After the cells were seeded, they were incubated for 48 h in medium without FBS with a range of concentrations of 3 to 200 µg/mL amoxicillin. The results showed that amoxicillin significantly and dose-dependently lowered *ApoA-I* expression (maximum = −30%). This lower mRNA expression also translated into a lower ApoA-I protein secretion in the culture medium. This indicates that, besides the potential indirect effects mediated via lower SCFAs formation, there is also a direct inhibitory effect of amoxicillin on both ApoA-I transcription and secretion. Interestingly, effects were specific for amoxicillin and could not be shown for penicillin and streptomycin. From a mechanistic point of view, it was shown that *KEAP1* and *CPT1* expressions were reduced by amoxicillin treatments. In addition, amoxicillin lowered the PPARα transactivation in HepG2 cells as well. As suggested by the authors, this points towards involvement of PPARα transactivation in the direct effects of antibiotics on ApoA-I expression and secretion, whereas a role for ER stress and BET inhibition seems not implicated [64]. In Figure 2, a schematic representation of our review is provided.

## 5. Limitations and Future Directions

Unfortunately, most human intervention studies in which the effects of dietary fibres were evaluated only analysed serum HDL-C, but not ApoA-I concentrations [8,30]. Nevertheless, some studies compared for example the effects of a high fibre diet to a low fibre diets, with ApoA-I concentrations as an endpoint. Smolders et al. evaluated the available literature and concluded that there were no differences in fasting ApoA-I concentrations [30]. Other studies, for example a high β-glucan and psyllium diet as compared with a low fat, low-cholesterol control diet, even showed reduced fasting ApoA-I levels [30]. So, current evidence is clearly not supportive to conclude that high fibre diets translate into higher plasma ApoA-I concentrations. Although these studies evaluated possible effects of fibres on fasting ApoA-I concentrations, the focus of these studies was not to find a fibre that increased SCFAs. Therefore, it is very well possible that this was not the right type of fibre or experimental setup when aiming at elevated SCFAs and consequent ApoA-I production. Using another type of fibre could have led to different outcomes in the ApoA-I levels. The results included in our review suggest that SCFAs, butyrate in particular, are able to increase *ApoA-I* mRNA levels in HepG2 cells. Therefore, there is a clear need for well-controlled human intervention studies evaluating the effects of a dietary fibre that stimulates for example intestinal butyrate production in which changes in ApoA-I production are explored as well. In this context, one could for instance think of inulin which belongs to the fructans, a group of non-digestible carbohydrates, that have shown to result in increased butyrate concentrations [65,66,67]. 

## 6. Conclusions

ApoA-I concentrations could potentially be elevated via a higher intake of dietary fibres, which enhances the production of SCFAs in the intestinal lumen and consequent amount of SCFAs entering the liver via the portal vein. SCFAs, especially butyrate, seem able to increase hepatic *ApoA-I* mRNA expression. The full overview of underlying mechanisms which are responsible for this higher *ApoA-I* mRNA expression still needs to be explored in more detail, but at least BET inhibition and PPARα activation seem to be involved. Future research should focus more on the possible cross-talk between the intestine and liver. Finally, there is a need for well-controlled intervention studies with specific dietary fibres that elevate SCFAs production in the intestinal lumen and translates to an increase in newly produced ApoA-I in the liver.
